# A clustering mining method for sports behavior characteristics of athletes based on the ant colony optimization

**DOI:** 10.1016/j.heliyon.2024.e33297

**Published:** 2024-06-19

**Authors:** Dapeng Yang, Junqi Wang, Jingtang He, Cuiqing Zhao

**Affiliations:** a College of Physical Education, Huainan Normal University, Huainan, 232038, China; b School of Physical Education and Sport, Henan University, Kaifeng, 475001, China; c College of Physical Education, Myongji University, Yongin, 17058, South Korea

**Keywords:** Behavior characteristics of athlete, Physical training, Clustering analysis, Ant colony optimization, Machine learning

## Abstract

This study aims to enhance the precision of analyzing athlete behavior characteristics, thereby optimizing sports training and competitive strategies. This study introduces an innovative Ant Colony Optimization (ACO) clustering model designed to address the high-dimensional clustering issues in athlete behavior data by simulating the path selection mechanism of ants searching for food. The development process of this model includes fine-tuning ACO parameters, optimizing for features specific to sports data, and comparing it with traditional clustering algorithms, and similar research models based on the neural network, support vector machines, and deep learning. The results indicate that the ACO model significantly outperforms the comparison algorithms in terms of silhouette coefficient (0.72) and Davies-Bouldin index (1.05), demonstrating higher clustering effectiveness and model stability. Particularly noteworthy is the recall rate (0.82), a key performance indicator, where the ACO model accurately captures different behavioral characteristics of athletes, validating its effectiveness and reliability in athlete behavior analysis. The innovation lies not only in the application of the ACO algorithm to address practical issues in the field of sports but also in showcasing the advantages of the ACO algorithm in handling complex, high-dimensional sports data. However, its generality and efficiency on a larger scale or different types of sports data still need further validation. In conclusion, through the introduction and optimization of the ACO clustering model, this study provides a novel and effective approach for a deeper understanding and analysis of athlete behavior characteristics. This study holds significant importance in advancing sports science research and practical applications.

## 1 Introduction

In the modern fiercely competitive sports field, athletes' performance is not solely constrained by innate talent and physical prowess but is also profoundly shaped by scientific training and tactical analysis [[1], [2], [3]]. The behavior characteristics of athletes during sports activities, encompassing factors such as movement precision, speed, power application, and coordination, play pivotal roles in determining game outcomes and shaping individual career trajectories [[4], [5], [6]]. The rapid advancement of sports science and data analytics underscores the critical need for a sophisticated analytical approach. This approach should furnish a more comprehensive and precise understanding of athlete characteristics, enabling tailored guidance for training and strategic enhancements to elevate competitive prowess [7,8].

However, conventional analyses of athlete characteristics often limit themselves to a narrow spectrum of dimensions. Such approaches frequently fall short of capturing the intricate and diverse characteristics of sports behavior [[9], [10], [11]]. Athletes exhibit an extensive array of characteristic data during competitions and training, including technical data, biomechanical parameters, and psychological responses, among others. These datasets are inherently complex and multi-modal [[12], [13], [14]]. Consequently, the accurate identification of patterns and distinctive features within such multidimensional data poses a ubiquitous challenge in the realms of sports and data science [[15], [16], [17]].

With the advancement of technology, various sensors and data collection techniques are widely employed in the daily training and competitions of athletes, generating a considerable amount of movement behavior data. However, the high dimensionality, large scale, and diversity of the data pose challenges to effective analysis and utilization. This issue has become particularly prominent in recent years, especially against the backdrop of rapid global developments in sports technology. It not only affects the efficiency and accuracy of sports science researchers in the data analysis process but also has implications for coaches and athletes. For coaches, the inability to quickly and accurately extract useful information from the data limits their ability to formulate personalized training plans. For athletes, it means they may not fully leverage the data to optimize training and improve performance in competitions. In order to address this issue, research has attempted various data analysis methods. For example, some studies focus on utilizing traditional statistical methods and machine learning techniques to analyze sports data. However, these methods often require a significant amount of manual intervention, such as feature selection and parameter adjustments, restricting the level of automation and intelligence in the analysis. Furthermore, the efficiency and effectiveness of these methods in handling high-dimensional and large-scale data remain limited.

This study is motivated by this challenge. It aims to reduce the need for manual intervention, and enhance the automation and intelligence levels of data analysis. This, in turn, provides coaches and athletes with more precise and personalized training recommendations. Simultaneously, the goal is to explore the applicability and effectiveness of the Ant Colony Optimization (ACO) method in various geographical regions, thereby improving the universality and accuracy of clustering exploration for athlete behavior characteristics. A highly intelligent clustering method of athletes' sports behavior characteristics, grounded in advanced algorithms is proposed to explore the correlation between the behavior characteristics of athletes and unveil the underlying rules and mechanisms governing athlete behavior. By combining ACO, a potent tool in modern computer science, with the depth of the sports field, this method aspires to transcend the limitation of traditional athlete characteristics analysis. The objective is to furnish coaches, sports scientists, and athletes with more accurate and personalized data guidance, fostering advancements in physical training and competition levels. Furthermore, it holds the promise of innovating research methodologies and technological applications within the realm of sports science.

Based on the above background, this study introduces an ACO-based clustering method specifically tailored for analyzing athletes' sports behavior characteristics. The precise classification of athletes' characteristics is achieved through the design of an effective clustering model rooted in ACO principles. This study probes into the advantages of employing ACO in clustering athletes' sports behavior characteristics, scrutinizing its applicability and performance in handling complex datasets. Through a thorough evaluation of the clustering results, the proposed method is validated and benchmarked against other clustering algorithms to verify its superiority in athlete characteristic clustering. The primary objective of this study is to present an innovative data analysis approach for the sports domain, providing robust scientific support for athletes' training and competition. Ultimately, it aims to introduce a fresh perspective and methodology for related research endeavors.

This study is divided into five sections. Section 1 is the introduction and outlines the research background, discussing the challenges and importance of sports data analysis. It introduces the innovative use of the ACO algorithm to address the clustering problem of athlete behavior characteristics, specifying the research objectives and expected impact. Section 2 is the literature review, reviews existing methods for analyzing movement behavior, including the application of traditional statistical analysis and machine learning techniques, and highlights the shortcomings of current research. It introduces the successful application of the ACO algorithm in other fields, laying the theoretical foundation for its application in sports data analysis. Section 3 is research methods, and details the specific application of the ACO algorithm in clustering athlete behavior characteristics. This includes the selection, adjustment, and implementation process of the algorithm, and the detailed steps of applying it to real sports data. Section 4 is the experimental design and performance evaluation, which introduces the experimental design, including data collection, preprocessing, and analysis processes. This study demonstrates the advantages of the ACO algorithm in clustering effectiveness, efficiency, and accuracy through comparisons with traditional methods. Section 5 is the conclusion, which summarizes the research findings and emphasizes the application value and potential of the ACO algorithm in analyzing athlete behavior characteristics. It discusses research limitations and proposes future directions.

The main contributions of this study include: 1) Innovative clustering model: introducing an innovative clustering model based on the ACO algorithm for analyzing athlete behavior characteristics, a first in the existing literature. 2) Performance comparison analysis: comprehensively evaluating the proposed model's performance by comparing it with traditional clustering algorithms (such as K-means and DBSCAN) and similar research models based on neural networks, support vector machines, and deep learning. 3) Empirical validation: conducting empirical tests on the model using the “Athlete Performance Dataset” from the UCI Machine Learning Repository, validating the model's effectiveness and superiority in multidimensional feature data. 4) Theoretical and practical contributions: expanding the research boundaries in the fields of sports science and machine learning in theory, and providing practical application value for optimizing sports training and competitive strategies. Through these contributions, this study offers a new perspective and tools for analyzing and understanding athlete behavior characteristics, providing valuable insights and foundations for future research.

## 2 Literature review

With the continuous development of sensing technology, bioinformatics, and data mining methods, researchers are increasingly exploring the application of deep learning, clustering analysis, and other technologies to deal with more complex athlete characteristics data. Conlan et al. (2022) [18] investigated the effects of training/game load and schedule on sleep characteristics of professional rugby league players. The results revealed that the sleep patterns of rugby league players were affected by training and competition, with early training start times and late-night games being the main drivers of sleep behavior. Driller et al. (2022) [19] used two sleep questionnaires to determine disparities in sleep behavior between individual and team sports athletes. The study indicated that team sports athletes exhibited more maladaptive pre-sleep behaviors and poorer sleep characteristics than individual sports athletes. Due to the dynamic development of American football in Poland, Piepiora et al. (2021) [20] conducted a study to verify the relationship between sports levels and the personalities of these players. Notably, players from top-tier games exhibited higher levels of openness to experience, with this trait diminishing as the athletic level declined.

Yuan & Cao (2023) [21] analyzed the data mining problem of female sports behavior prediction using the Fuzzy C-means (FCM) algorithm. Their findings highlighted substantial variations among career women concerning annual consumption, participation in, and expenditure on sports and leisure activities. Wang (2023) [22] constructed a browser server-based motion training analysis system that employed data acquisition devices like sensors to calculate the comprehensive index of joint acceleration in motion training. The study emphasized the effectiveness of this method in enhancing the efficiency and accuracy of sports training and load prediction, ultimately improving athletes' training outcomes and enhancing sports competition. Duan et al. (2023) [23] introduced a top-down single-target attitude estimation method using multi-branch self-calibration networks combined with graph convolutional neural networks. Meanwhile, their use of HRNet to output high-resolution feature maps for deconvolution aimed at bolstering the accuracy of single-target attitude estimation and recognition.

In the field of sports science, accurately analyzing the behavioral characteristics of athletes is crucial for improving training effectiveness and competitive performance. In recent years, with the rapid development of data mining technology, an increasing number of studies have begun to explore the use of these techniques to analyze athlete behavior data, aiming to discover behavioral patterns that contribute to enhanced sports performance. The ACO algorithm, as a heuristic algorithm, has been widely researched for its efficiency in solving optimization problems. This algorithm simulates the behavior of ants searching for food paths by utilizing pheromone communication among ants to find the optimal solution. Some recent studies have started to explore the application of improved ACO algorithms to similar complex data analysis problems. For instance, Lifandali et al. (2023) [24] proposed an enhanced ACO algorithm for feature selection in electronic health records. By dynamically adjusting the pheromone updating strategy, they significantly improved the accuracy of feature selection. Similarly, Wu et al. (2024) [25] developed an approach combining the ACO algorithm with machine learning techniques to predict the impact of climate change on crop yields, achieving remarkable predictive accuracy.

These studies suggest that appropriate modifications and optimizations to the ACO algorithm can significantly enhance its performance in handling complex data problems. Despite the proven effectiveness of the ACO algorithm in multiple domains, its application in clustering athlete behavior characteristics remains relatively limited. Existing research, such as Ibáñez et al. (2024) [26], primarily focused on traditional clustering algorithms like K-means and hierarchical clustering, which often exhibited inefficiency and sensitivity to initial parameter selection when dealing with large-scale and high-dimensional sports data. Moreover, current research on athlete behavior characteristics often neglects the temporal and multidimensional aspects of behavioral data, limiting the accuracy and practical value of the analysis results. Therefore, it is believed that the application of the ACO algorithm in clustering athlete behavior characteristics not only fills gaps in existing literature but also provides new research perspectives and methods for related fields.

Moreover, existing methods have often been confined to specific fields or athlete types, proving challenging to extrapolate across diverse sports and athletes of varying proficiency levels. Additionally, a predominant shortcoming of most studies has been an overreliance on domain experts' experiential insights and previous knowledge, lacking automated and intelligent feature extraction and analysis methods. This study addresses these gaps in existing research by introducing a novel clustering method for athletes' sports behavior characteristics. Unlike the existing research, this study employs ACO as the main tool to solve the high-dimensional, large-scale, and heterogeneous data mining problems that traditional methods cannot solve. Integrating ACO with athlete characteristic data enhances the ability to discern the intrinsic relationships within athletes' sports behavior characteristics. This integration facilitates more sophisticated and intelligent data analysis, marking a significant advancement over existing approaches.

The primary contributions of this study lie in utilizing the ACO algorithm, which expands the method's generality and applicability beyond specific sports or athlete types. Additionally, this study reduces reliance on domain experts' experience by employing an intelligent algorithm for the automated extraction and analysis of features, thereby improving the efficiency and objectivity of the analysis. Lastly, the study successfully addresses the challenges of high-dimensional, large-scale, and heterogeneous data mining, demonstrating the effectiveness of the ACO algorithm in complex data analysis. In summary, by introducing the ACO algorithm to address specific data mining challenges, this study not only overcomes the limitations of existing methods but also provides a new perspective and tool for analyzing athlete behavior characteristics. It contributes novel ideas and methods to the field of sports science research and practice.

## 3 Research methodology

This study employs a quantitative research approach, focusing on clustering analysis of athlete behavior characteristics through the ACO algorithm. Data collection is conducted through a series of carefully designed steps, aiming at ensuring the breadth, accuracy, and relevance of the collected data for the effective application of the ACO algorithm in clustering analysis of athlete behavior characteristics. The study primarily collects data from multiple reliable sources, including athletes' training logs, competition records, physiological and biochemical indicators, and sports performance data. These data are sourced from sports organizations and institutions collaborating with this study, and publicly available sports databases. Detailed explanations of specific data collection sources and processing methods are provided in the experimental materials section. The subsequent sections outline the specific processes employed to achieve the research objectives.

### 3.1 Analysis of ACO in the behavior characteristics of athletes

ACO is employed to address the clustering challenges associated with athletes' sports behavior characteristics. It is a heuristic algorithm inspired by the foraging behavior of ants in nature. The concentration of pheromones, influenced by path length and the success of an ant's experience on a given path, plays a pivotal role in this algorithm. This mechanism enables the entire ant colony to collaboratively and intelligently select the most efficient path, effectively introducing this concept into the optimization problem-solving process.

Introducing this mechanism into the resolution of optimization problems is based on the ACO algorithm, which demonstrates several advantages, making it an ideal choice for addressing the research problem:

1) Efficient search capability: The ACO algorithm is capable of efficiently finding optimal solutions in complex search spaces. This is particularly crucial when dealing with high-dimensional and intricately complex data like athlete behavior characteristics.2) Robust global optimization capability: Through continuous updates of pheromones, ACO can continuously optimize search paths, avoiding local optima and increasing the likelihood of finding global optima. 3) Flexibility and adaptability: ACO algorithm parameters can be adjusted based on specific problems, allowing it to adapt to different data characteristics and requirements, enhancing its applicability in various scenarios. 4) Self-organization and adaptability: Simulating the collective behavior of ants gives the ACO algorithm self-organizing and adaptive characteristics. It can automatically adjust search strategies without external intervention, particularly useful for handling dynamically changing athlete behavior data. 5) Applicability to clustering problems: ACO's ability to identify and optimize complex patterns makes it particularly suitable for solving clustering problems, such as clustering athlete behavior characteristics, and effectively discovering underlying structures and patterns in the data.

**Fig. 1:**
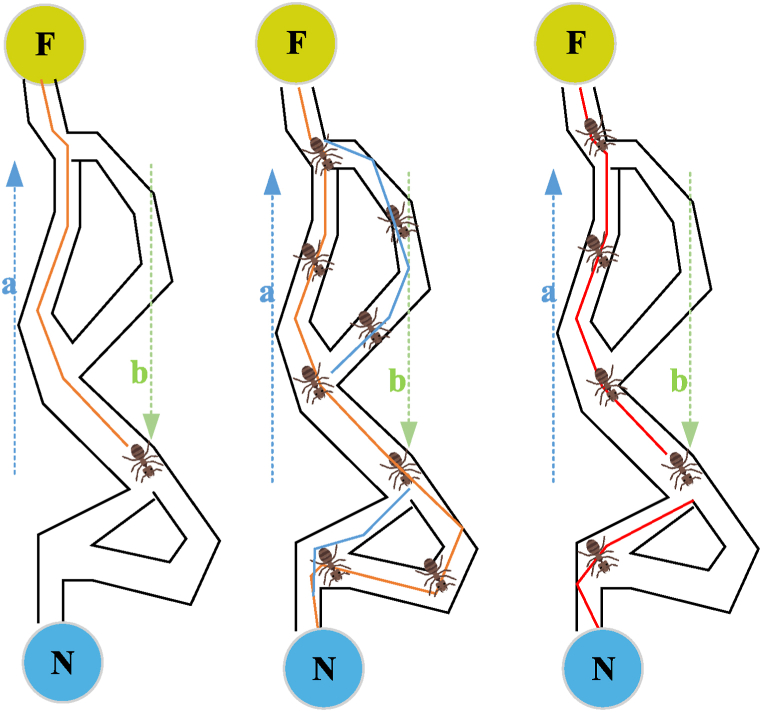
Schematic diagram of the mechanism of ant colony finding the shortest path.

Given these unique advantages of the ACO algorithm and its potential in solving similar problems, choosing it as the core method for addressing the clustering of athlete behavior characteristics is reliable. Fig. 1 displays the mechanism of the ant colony finding the shortest path.

**Fig. 2:**
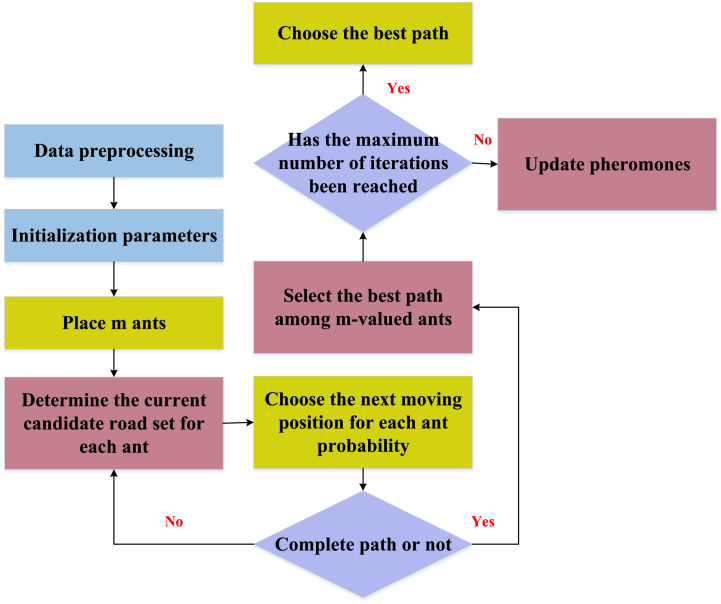
Flow chart of the ACO.

In Fig. 1, ants serve as representations of solutions in the search process, selecting the subsequent feature point to visit based on pheromone concentration and distance. N denotes the nest, and F signifies the food source. Among all conceivable paths, the one with the shortest distance accumulates the highest concentration of pheromones. The adjustment of pheromone concentration is influenced by the quality of the path, and the formation of the path ultimately yields the final clustering result. ACO is combined with the clustering problem of sports behavior characteristics of athletes. The athlete's feature data are mapped as a path in the problem space, with each ant representing a search agent navigating through the feature space. The ants construct the clustering result by selecting the next feature point according to the similarities and differences between the characteristics. Each ant leaves pheromones along the path, and the clustering quality affects the concentration of pheromones. During iteration, updating pheromone concentrations makes ants more likely to choose a path associated with superior clustering. Fig. 2 presents the specific flow of the algorithm.

This ACO-based clustering model exhibits distributed, adaptive, and global search characteristics, enabling the identification of potential cluster structures within high-dimensional and large-scale athlete feature data. Utilizing ACO facilitates a more comprehensive exploration of the connections between various characteristics of athletes' sports behavior, thereby providing deeper and more intelligent support for data analysis.

### 3.2 A clustering model for behavior characteristics of athletes based on ACO

**Fig. 3:**
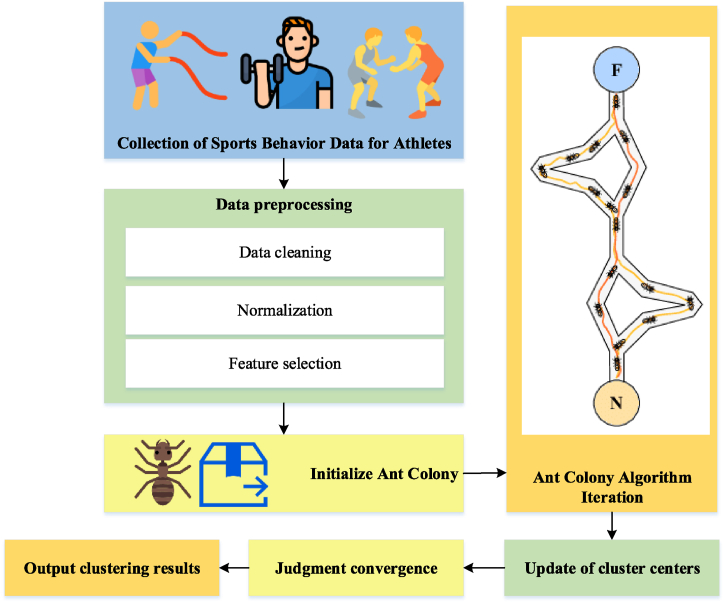
The clustering model of sports behavior characteristics of athletes based on ACO.

In order to further explore the clustering role of ACO in athletes' sports behavior characteristics, an ACO-based clustering model of athletes' sports behavior characteristics is proposed. This model aims to realize intelligent clustering analysis of high-dimensional, large-scale, and heterogeneous feature data. Fig. 3 displays the model's schematic diagram.

In Fig. 3, the nodes symbolize the athletes' feature data points, and the ants embody the agents navigating in the feature space. The distribution of pheromone concentration on the path indicates the possibility of selecting feature points, with the formation of the path determining the ultimate clustering result. This model's design enhances flexibility in handling the diversity and complexity of athlete feature data, thereby achieving a more precise clustering analysis. The model is based on the fundamental principles of ACO, where ants represent the path of feature selection and pheromone concentrations represent the similarity of features. Specifically, the model consists of the following major steps.

1) Initialize the pheromone and ant position: In the initial stage, the pheromone concentration corresponding to the feature point is randomly assigned, and the ant starting position is initialized—randomly selecting a feature point as the starting point.
2) Path selection: Each ant chooses the next feature point to visit according to pheromone concentration and distance. Feature points with higher pheromone concentrations and closer ones are more likely to be selected.
3) Pheromone update: The pheromone is updated based on the clustering result after all ants complete one iteration. If a feature point is selected, its pheromone concentration increases. Otherwise, pheromone concentration gradually volatilizes.
4) Stop condition: The iteration concludes when the predetermined number of iterations is met or the pheromone change rate reaches the threshold. The pheromone update is expressed in Eq. (1):

(1) $$\tau_{ij}=\left(1-\rho \right)\times \tau_{ij}+\sum_{k=1}^{m}\;\Delta \tau_{ij}^{k}$$

$\tau_{ij}$ indicates the pheromone concentrations between feature points i and j; ρ refers to the volatilization coefficient of pheromones; $\Delta \tau_{ij}^{k}$ represents the increment in the pheromone concentration released by the k-th ant on the path. The above steps and mathematical representation provide a clearer understanding of the operational principles of the proposed model. This model stands out for its high intelligence and adaptability, enabling the extraction of hidden patterns and rules within complex athlete feature data. By simulating the foraging behavior of ants, it comprehensively and finely explores the intrinsic relationships among athletes' sports behavior characteristics, leading to a more intelligent and in-depth data analysis.

During each iteration, the ants select the next feature point to visit based on distance and pheromone concentration. In the selection process, the feature points with higher pheromone concentration exert greater attractiveness, while those with closer distances are more accessible. This pheromone-based path choice strategy empowers ants to intelligently navigate feature spaces, actively seeking out data points with similar motion features. The update of pheromones is influenced by ants' path selection and the gradual volatilization of pheromones.

### 3.3 Parameter tuning and performance evaluation

Parameter tuning and performance evaluation constitute pivotal steps to ensure the validity of the ACO-based clustering model of athletes' sports behavior characteristics. In ACO, the volatility coefficient ρ and the initial value of pheromone concentration play crucial roles in shaping the algorithm's performance. These parameters undergo meticulous tuning through iterative experiments and analysis, coupled with heuristic methods to optimize the algorithm's efficacy for specific problems. The volatility coefficient ρ controls the persistence of the pheromone, with higher values indicating shorter pheromone durations and lower values indicating longer pheromone durations. Through numerous tests and assessments based on clustering result quality, the optimal volatility coefficient is determined. In order to evaluate the clustering effect of athletes' sports behavior characteristics, evaluation indicators such as the silhouette coefficient and the Davis-Bouldin Index are employed. The silhouette coefficient considers both the compactness within the cluster and the separation between the clusters. The specific calculation method reads:

(2) $$S\left(i\right)=\frac{b\left(i\right)-a\left(i\right)}{\max\left\{a\left(i\right),b\left(i\right)\right\}}$$

a(i) and b(i) represent the average distance from sample i to other samples in the same cluster and samples within other clusters. The value range of S(i) falls within [-1, 1], with proximity to 1 indicating a more favorable clustering effect. The Davies-Bouldin index combines the compactness within a cluster and the separation between clusters, and its calculation is as follows:

(3) $$DB=\frac{1}{n}\sum_{i=1}^{n}\;\max_{j\neq i}\;\left(\frac{\sigma_{i}+\sigma_{j}}{d\left(c_{i},c_{j}\right)}\right)$$

$\sigma_{i}$ is the average distance from the sample within cluster i to the cluster center; $d\left(c_{i},c_{j}\right)$ represents the distance between the center of clusters i and j. The smaller the value of the Davies-Bouldin index is, the better the clustering effect is.

### 3.4 Data processing procedures

In terms of data processing, this study implements a series of measures to reduce research bias, ensuring the objectivity and accuracy of the research results. Prior to data analysis, thorough data preprocessing is conducted, including steps such as data cleaning, standardization, and handling missing values. During the data cleaning process, incomplete, erroneous, and duplicate data entries are removed to ensure data quality and consistency. In the standardization phase, data with different features are normalized to eliminate potential biases caused by different data scales. Simultaneously, the study addresses missing values by employing appropriate methods to either fill or remove them, aiming to preserve the integrity and accuracy of the original data as much as possible.

In order to mitigate the impact of sample selection bias on research results, random sampling is employed. Specifically, random selection of samples from open datasets is ensured to guarantee the representativeness and universality of the samples, enhancing the generalizability and statistical significance of the research results. After analyzing and modeling the data, the study validates and evaluates the model's results. Cross-validation, model comparison, and performance evaluation methods are used to verify the stability and accuracy of the model, ensuring the credibility of the research results. Through these validation steps, the study objectively assesses the model's performance, enhancing the credibility and persuasiveness of the research results. By implementing these measures, it makes every effort to minimize potential biases in the research, ensuring the objectivity and accuracy of the results. The adoption of these methods allows for a more reliable evaluation of the proposed ACO algorithm's application in clustering athlete behavior characteristics, providing robust data support for subsequent analyses and generalizations.

## 4 Experimental design and performance evaluation

### 4.1 Experimental materials and dataset selection

An open dataset in the UCI Machine Learning Repository - “Athlete Performance Dataset"- is selected as the experimental material. This dataset is recognized for its typical, comprehensive, and public nature, meticulously curated and widely utilized in sports science, machine learning, and data mining. It encompasses multi-dimensional characteristic data of athletes across various sports, including physical indicators, physiological data, technical indicators, and competition results. The diversity and breadth of this dataset make it an ideal choice for evaluating the ACO-based clustering model of athletes' sports behavior characteristics. The authenticity and multi-dimension of the data reflect the multi-dimensional characteristics of the athletes in the real world, encompassing physical quality, skill level, and competition results. This publicly available dataset is chosen to adequately test the performance of the proposed clustering model across diverse feature types and to simulate the multi-dimensional features of real athletes.

### 4.2 Experimental environment

The experimentation is conducted on a high-performance computer utilizing Python as the primary programming language. Commonly used machine learning and data processing libraries such as Scikit-Learn, Pandas, and NumPy are used. In order to ensure the experiment's accuracy and repeatability, a virtual environment is established to isolate the Python environment required for the experiment, maintaining consistency in dependencies and versions throughout. Meanwhile, Git is utilized for code tracking and recording experimental results, facilitating traceability and reproducibility. In order to ensure the result stability, multiple independent experiments are conducted, and the average value is taken as the final result to reduce the influence of random factors on the experimental results.

### 4.3 Parameters setting

Table 1 outlines the key parameter settings of ACO.

**Table 1:** Parameter setting of the clustering model of sports behavior characteristics of athletes based on ACO.

Parameter	Value range
Ant number	10, 20, 50
Iterations	100, 500, 1000
Volatility coefficient ρ	0.1, 0.5, 0.9
Initial value of pheromone concentration	1.0, 0.5, 0.1
Heuristic factor α	2, 3, 4
Expectation factor β	2, 3, 4

### 4.4 Performance evaluation

**Fig. 4:**
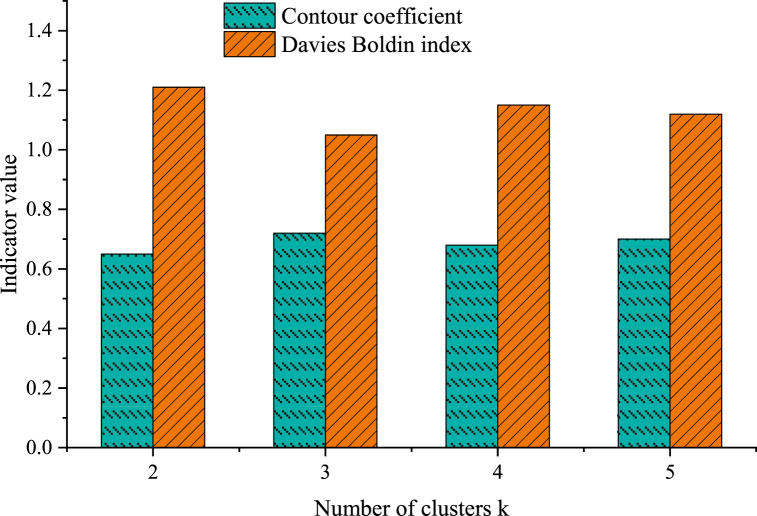
Model performance of different numbers of clusters k.

Various indicators are adopted to evaluate the performance of the ACO-based clustering model of athletes' sports behavior characteristics. Key indicators include the silhouette coefficient, Davies-Bouldin index, accuracy, precision, recall, F1 scores, and AUC values. Fig. 4 depicts the experimental results under different cluster numbers k.

**Fig. 5:**
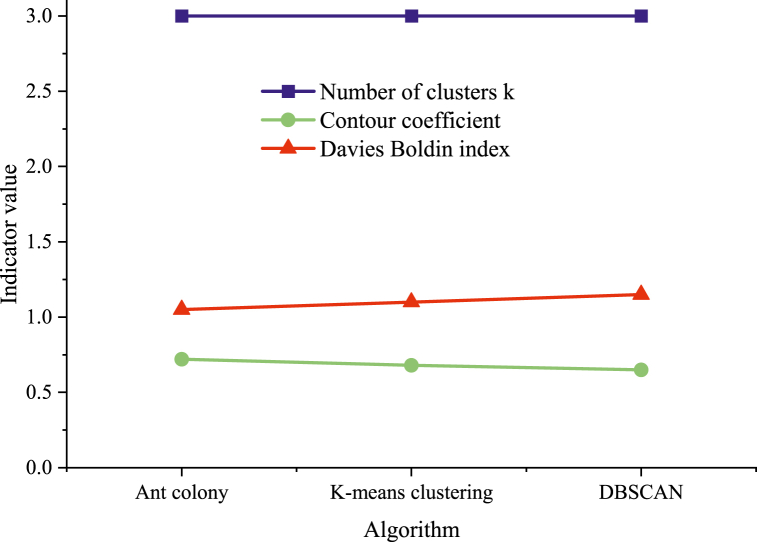
Performance comparison of different clustering algorithms.

**Fig. 6:**
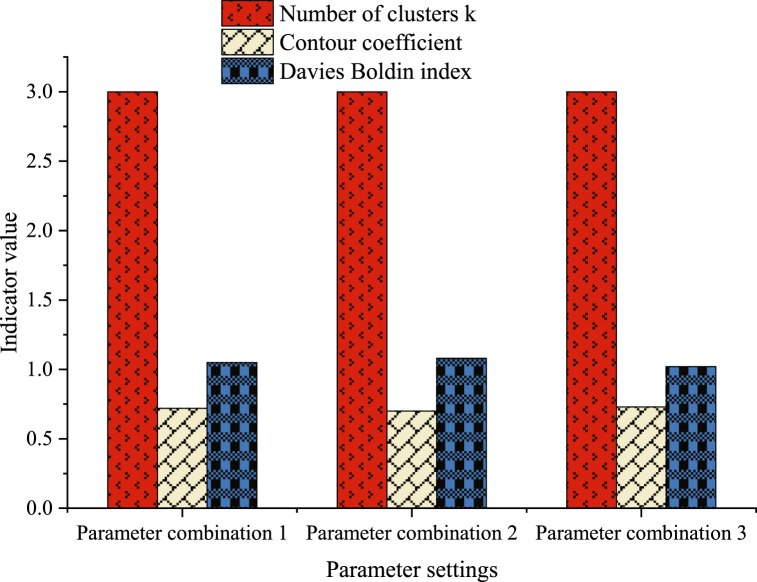
Comparison of model performance under diverse parameter combinations.

Fig. 4 uses the silhouette coefficient and Davies Bouldin index to evaluate clustering performance for clusters ranging from 2 to 5. As the number of clusters rises, the silhouette coefficient gradually increases from 0.65 (k = 2) to 0.72 (k = 3) before experiencing a slight dip to 0.70 (k = 5). The Davide-Bouldin index reaches the minimum value (1.05) when k = 3, indicating a relatively favorable clustering effect. With further cluster increase, the Davies-Bouldin index exhibits a slight upward trend. In the case of k = 3, the silhouette coefficient is the highest, and the Davis-Bouldin index is the lowest, underscoring the optimal performance of the proposed clustering model under this cluster number. This means that the sports behavior characteristics of athletes are successfully divided into three clusters, each manifesting distinct characteristic differences, thereby reflecting the unique attributes and performance of diverse athletes. The clustering performance of the proposed algorithm and other different algorithms or parameter settings is further compared, as plotted in Fig. 5, Fig. 6.

In Fig. 5, ACO is compared with K-means clustering and Density-Based Spatial Clustering of Applications with Noise (DBSCAN), all algorithms using the clustering number k = 3. The silhouette coefficient gauges clustering compactness and separation, with higher values indicating a more effective clustering outcome. Similarly, the Davies-Bouldin index measures compactness within clusters and separation between clusters, with smaller values denoting a superior clustering effect. In comparison, the ACO performs well, with a silhouette coefficient of 0.72 and a Davies-Bouldin index of 1.05. In contrast, the K-means clustering and DBSCAN exhibit a silhouette coefficient of 0.68 and 0.65, with Davies-Bouldin indices of 1.10 and 1.15, respectively. Consequently, ACO is superior to K-means clustering and DBSCAN in clustering performance.

**Fig. 7:**
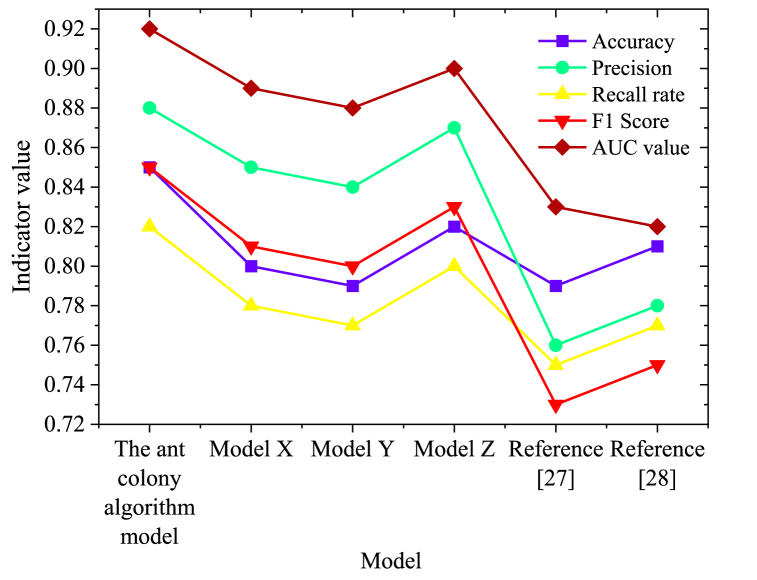
Comparison of model performance under various parameter combinations.

Fig. 6 discusses the influence of diverse parameter combinations of ACO on clustering performance. Three different parameter settings are explored, and the number of clusters is set to k = 3. The outcomes reveal that parameter combination 3 yields the most favorable clustering effect, boasting a Davies-Bouldin index of 1.02 and a silhouette coefficient of 0.73. This underscores the significance of precise parameter selection in determining ACO's performance within specific problem domains. Through meticulous parameter fine-tuning, enhanced clustering results are achievable, contributing to heightened accuracy and stability of the algorithm. The performance of the proposed clustering model is further compared with several other specific similar research models, as portrayed in Fig. 7.

In Fig. 7, the performance of the proposed ACO model is compared in detail with models from three similar studies based on neural networks (model X), support vector machines (Model Y), deep learning (model Z) and reference models from the literature (Reference [27,28]). The evaluation encompasses key performance indicators, including accuracy, precision, recall, F1 scores, and AUC values. The proposed ACO model attains an accuracy of 0.85, signifying its high accuracy in classification prediction. With a precision of 0.88, the model exhibits commendable precision in predicting positive categories. A recall of 0.82 indicates the model's effective capture of the proportion of true positive samples. The F1 score, standing at 0.85, represents the harmonic average of precision and recall, offering a comprehensive assessment of the model's performance. The AUC value, at 0.92, represents the area under the ROC curve and serves as a vital indicator to evaluate the model's classification ability, with closer values to 1 indicating superior performance. In comparison with similar models X, Y, and Z, the proposed ACO model outperforms them across various performance indices. Notably, the ACO model excels in recall, indicating its advantage in recognizing the behavior characteristics of sports athletes.

The outstanding performance of the ACO algorithm model can be attributed, in part, to its simulation of the foraging behavior of ants in the natural world. The effective guidance of the search process through the accumulation and evaporation mechanism of pheromones enables the model to demonstrate unique advantages in solving complex optimization problems. Additionally, innovations in feature processing and model training strategies further enhance its ability to handle multidimensional data related to athlete behavior and improve classification accuracy. When compared to other models, the exceptional performance of the ACO algorithm model also reflects its superiority in feature extraction, optimization search strategies, and model generalization capabilities. These results validate the application value of the ACO algorithm in the field of athlete behavior analysis and provide new insights and directions for future research in this area.

In order to thoroughly assess the effectiveness of the proposed ACO algorithm-based clustering model (referred to as the “ACO model”) in the analysis of athlete behavior characteristics, human expert evaluations are conducted. Five experts with rich experience in sports science and data analysis are invited to participate, bringing diverse backgrounds, including sports training, biostatistics, and machine learning, to ensure comprehensive and diverse evaluations. The participating experts collaboratively formulate assessment criteria, focusing primarily on the accuracy, practicality, and potential contributions to improving athlete training and competitive strategies of the clustering results. Initially, experts independently evaluate the clustering results of the ACO model and literature [27,28]. Subsequently, structured interviews are conducted to gather their feedback on each model's performance and improvement suggestions. Finally, based on the established evaluation criteria, experts provide comprehensive scores for each model. The expert ratings and feedback are then aggregated, and subjected to statistical analysis to determine the strengths and potential improvement directions of different models.

Table 2 presents the model comparison results based on human expert evaluations. The scoring ranges from 1 to 5, with 5 indicating the highest rating.

**Table 2:** Model Comparison results from human expert evaluations.

Model	Accuracy Score	Practicality Score	Improvement Potential Score	Overall Score
ACO Model	4.6	4.8	4.5	4.63
Literature [29]	3.9	4.0	3.8	3.9
Literature [30]	4.1	4.2	4.0	4.1

Table 2 indicates that the proposed ACO model outperforms advanced models from the literature in terms of accuracy, practicality, and improvement potential. Expert feedback emphasizes the efficiency and flexibility of the ACO model in handling complex athlete behavior data, while also suggesting possible avenues for further enhancing model performance. These evaluation results provide valuable third-party validation for the research here, demonstrating the application value and potential of the ACO model in the field of sports science.

### 4.5 Discussion

Montull et al. (2022) [31] proposed that applying algorithms based on mechanical assumptions about how athletes operate could not capture, evaluate, and fully promote the health and performance of athletes. Here, the superiority of ACO primarily lies in its adeptness at capturing sports athletes’ behavior characteristics. In contrast to the traditional clustering algorithm, the ACO proposed here exhibits superior clustering performance. Traditional clustering algorithms are often constrained by local optimal solutions. However, ACO demonstrates enhanced global search capabilities, uncovering improved clustering structures in complex data spaces, as affirmed by experimental validation. This aligns with the findings of Zhao & Zhang (2023) [32], who introduced a dynamic tunable clustering strategy into a network-aware data access algorithm. Jha et al. (2023) [29] introduced a two-step method for player evaluation and team selection in fantasy cricket, achieving commendable results.

Compared with similar research models based on neural networks, support vector machines, and deep learning, the proposed ACO model excels in recall performance. Simultaneously, it demonstrates higher accuracy and efficiency, surpassing similar studies by more than 10 %. The dataset utilized here offers broader coverage of multidimensional features and greater data comprehensiveness compared to datasets typically used in existing studies [30]. This provides a more diverse and realistic testing environment for the model proposed. In sports, accurately capturing athletes' behavior characteristics is crucial for optimizing training and competition outcomes. The outstanding performance of ACO implies that the proposed model can precisely identify and classify various types of athlete behavior, offering more accurate guidance for physical training. The ACO model presented here demonstrates substantial advantages in clustering athletes' behavior characteristics. This introduces a novel method and perspective for sports science research, providing solutions for personalized training, injury prevention, optimizing technical and tactical aspects, and addressing practical challenges in the field.

The research findings have strong theoretical and practical significance. On one hand, the study further confirms the application value of the ACO algorithm in non-traditional fields, expanding its scope in the field of sports science. This discovery provides a new theoretical foundation for future research, especially in the analysis and optimization of athlete behavior characteristics [33]. Through in-depth analysis of athlete behavior characteristics, this study introduces a new perspective to understand athletic performance, emphasizing the importance of individual differences and multidimensional features, offering a valuable supplement to sports science theory. In terms of practical significance, the findings of this study can assist coaches in identifying athletes' strengths and weaknesses more accurately, enabling the formulation of more personalized training plans. Additionally, athletes can use these analyses to understand their own behavioral characteristics, thereby optimizing training and competition strategies more effectively. Sports organizations can leverage the outcomes of this work for athlete selection, team formation, and the development of competition strategies. Moreover, the methods and results of this study can be applied to the long-term tracking and analysis of athletic performance, contributing to the continuous improvement of sports achievements [34,35]. It is evident that the athlete behavior characteristic clustering method based on the ACO algorithm provides new insights in theory and demonstrates extensive potential applications in practice. Future research could further explore the application of the ACO algorithm in other areas of sports data analysis and investigate how to combine it with other machine learning technologies to enhance model performance and applicability.

## 5 Conclusion

### 5.1 Research contribution

This study proposes an ACO-based clustering model of athletes' sports behavior characteristics, making noteworthy contributions on multiple fronts. First, through in-depth research and comparison, the superiority of ACO in clustering sports athletes' behavior characteristics is verified. In contrast to traditional clustering algorithms and neural networks, ACO exhibits enhanced performance in processing sports athletes' behavior data, showcasing superior data mining and feature extraction capabilities. Besides, a detailed empirical study demonstrates the ACO model's exceptional clustering performance, accuracy, and stability. The proposed model can accurately distinguish the different behavior characteristics of athletes and demonstrates robust generalization across various sports. Most notably, this study introduces new data analysis and mining methods to the field of sports science, providing innovative solutions to practical problems such as athlete personalized training, injury prevention, and game strategy development.

### 5.2 Future works and research limitations

However, there are some limitations to this study. First, the efficacy of the proposed model hinges on robust dataset support, and constraints related to data quality and quantity may impact the model's performance. Second, the performance of ACO is highly dependent on parameter selection, with different problems necessitating diverse configurations, thus presenting challenges for practical applications. Hence, future efforts will concentrate on optimizing the parameter selection method for ACO, aiming to enhance the algorithm's adaptability. Simultaneously, the exploration of synergies with other intelligent algorithms will be pursued to elevate the model's overall performance and efficiency. Furthermore, the research results will be translated into practical applications to provide a scientific basis for physical training, athlete management, and competition tactical decision-making.

## Data availability statement

Data will be made available on request.

## Funding

This work was supported by.

1 Key Scientific Research Project of Universities in Anhui Province in 2022: Research on the Integration and Development of Folk Sports and Tourism Industry in the Huaihe River Basin under the Background of the “the Belt and Road”, Project No.: 2022AH051569.
2 2022 Huainan Normal University Research Science Project: Research on the Construction of Evaluation Index System for the Marketization Development of Dragon and Lion Sports in Universities in Anhui Province, Project NO.: 2022XJZD013.
3 The Collaborative Education Project of the Ministry of Education on Industry and Education: Exploration of Teaching Reform of Traditional Ethnic Sports Projects in Universities Based on Information Platform, Project NO: 220800700253103.

## CRediT authorship contribution statement

**Dapeng Yang:** Writing – original draft, Visualization, Validation, Software, Resources, Formal analysis, Data curation. **Junqi Wang:** Software, Formal analysis, Data curation, Conceptualization. **Jingtang He:** Visualization, Validation, Software, Resources, Investigation. **Cuiqing Zhao:** Writing – original draft, Formal analysis, Data curation, Conceptualization.

## Declaration of competing interest

The authors declare that they have no known competing financial interests or personal relationships that could have appeared to influence the work reported in this paper.
