# Supplementary material for: A clustering mining method for sports behavior characteristics of athletes based on the ant colony optimization
Source: Heliyon. 2024 Jun 19;10(12):e33297. doi: 10.1016/j.heliyon.2024.e33297 (PMC11252961; doi:10.1016/j.heliyon.2024.e33297)
Supplement: Multimedia component 1 [file mmc1.docx]

**Questionnaire: Sports Behavior Characteristics of Athletes**

Hello! Thank you very much for participating in this survey. The purpose of this questionnaire is to collect the views and experiences of athletes on sports behavior characteristics. Through your feedback, we hope to gain a deeper understanding of athletes' attention to specific features in different sports events and to explore their views on relevant clustering mining methods. To ensure your right to know, please read the following statement:

**1) Personal information protection:** All personal information provided will be used only for this survey and research, and will be strictly confidential. Your name and personal identification information will not be used for other purposes.

**2) Anonymity:** This survey is anonymous, and your responses will be summarized and presented statistically. No specific information related to you personally will be made public.

**3) Voluntary participation:** Your participation is completely voluntary, and you can choose to suspend the investigation at any time. Leaving midway will not have any negative impact on you.

**4) Contact information:** If you choose to provide contact information, we will only use this information when further discussion or obtaining your consent is needed.

**The following basic information section can be filled in according to your situation:**

**Personal information (optional):**

1. Your name (optional):
2. Your age:
3. Your gender:
4. Your profession:

**The following are the formal question items. Please tick the content that you think meets.**

**1. Type of Athletes:**

- Amateurs
- Professional athletes
- School or university sports team members
- Other (please specify):

**2. Please list the main sports or activities you have participated in:**

- Athletics
- Football
- Swimming
- Basketball
- Tennis
- Other (please specify):

**3. What do you think are the most important sports behavior characteristics in your sports career? (Multiple options available):**

- Technical level
- Physical fitness
- Psychological quality
- Team collaboration ability
- Strategy and tactical application
- Reaction speed
- Other (please specify):

**4. How do you think sports behavioral characteristics' weight varies among sports events? Please assign a weight percentage to the following sports (the total should be 100%):**

- Athletics
- Football
- Swimming
- Basketball
- Tennis
- Other (please specify):

**5. Have you received specialized training on sports behavior characteristics in your physical exercise?**

- Yes
- No

**6. How do you think clustering mining of sports behavior characteristics is helpful for individual training and team collaboration?**

- Improving personal performance
- Improving team collaboration
- Having equal assistance
- Uncertain

**7. Which method do you prefer to analyze and apply sports behavior characteristics for clustering mining methods?**

- Data-driven method
- Expert experience-driven method
- Combining the two methods

**8. Do you tend to use technical means to monitor and improve sports behavior characteristics during training?**

- Using technical means (please specify specific technology)
- Not using technical means
- Uncertain

**9. Do you think individual differences will affect the clustering mining results of sports behavior characteristics?**

- Yes
- No
- Uncertain

**10. What do you think are the most challenging sports behavior characteristics in your sports career?**

- Technical level
- Physical fitness
- Psychological quality
- Team collaboration ability
- Strategy and tactical application
- Reaction speed
- Other (please specify):

**11. Are you willing to participate in future related research or experiments?**

- Yes
- No

**12. Do you have any other opinions, suggestions, or supplements regarding the athletes' sports behavior characteristics?**

Thank you very much for your participation! Your feedback is crucial for our research. If you are willing to further participate in our discussion, you can provide your contact information (optional):

1. Email：
2. Telephone number:

Thank you for your time and cooperation!

Date:
